# The prognostic utility of ^18^F-FDG PET parameters in lymphoma patients under CAR-T-cell therapy: a systematic review and meta-analysis

**DOI:** 10.3389/fimmu.2024.1424269

**Published:** 2024-09-02

**Authors:** Akram Al-Ibraheem, Ahmed Saad Abdlkadir, Dhuha Ali Al-Adhami, Mike Sathekge, Henry Hee-Seung Bom, Mohammad Ma’koseh, Asem Mansour, Hikmat Abdel-Razeq, Kamal Al-Rabi, Enrique Estrada-Lobato, Maysaa Al-Hussaini, Ismail Matalka, Zaid Abdel Rahman, Stephano Fanti

**Affiliations:** ^1^ Department of Nuclear Medicine and PET/CT, King Hussein Cancer Center (KHCC), Amman, Jordan; ^2^ School of Medicine, The University of Jordan, Amman, Jordan; ^3^ Department of Nuclear Medicine, University of Pretoria & Steve Biko Academic Hospital, Pretoria, South Africa; ^4^ Nuclear Medicine Research Infrastructure (NuMeRI), Steve Biko Academic Hospital, Pretoria, South Africa; ^5^ Department of Nuclear Medicine, Steve Biko Academic Hospital, Pretoria, South Africa; ^6^ Department of Nuclear Medicine, Chonnam National University Medical School (CNUMS) and Hospital, Gwangju, Republic of Korea; ^7^ Department of Medicine, King Hussein Cancer Center (KHCC), Amman, Jordan; ^8^ Department of Diagnostic Radiology, King Hussein Cancer Center (KHCC), Amman, Jordan; ^9^ Nuclear Medicine and Diagnostic Section, Division of Human Health, International Atomic Energy Agency (IAEA), Vienna, Austria; ^10^ Department of Pathology, King Hussein Cancer Center (KHCC), Amman, Jordan; ^11^ Department of Pathology and Microbiology, King Abdullah University Hospital- Jordan University of Science and Technology, Irbid, Jordan; ^12^ Department of Pathology, Ras Al Khaimah Medical and Health Sciences University, Ras Al Khaimah, United Arab Emirates; ^13^ Nuclear Medicine Department, Istituto di Ricovero e Cura a Carattere Scientifico (IRCCS) Azienda Ospedaliero—Universitaria di Bologna, Bologna, Italy; ^14^ Department of Medical and Surgical Sciences (DIMEC), Alma Mater Studiorum University of Bologna, Bologna, Italy

**Keywords:** CAR T-cell, chimeric antigen receptor, CAR T cell therapy, immunotherapy, systematic review, meta-analysis, molecular imaging, FDG

## Abstract

**Background:**

Chimeric antigen receptor (CAR) T-cell therapy has attracted considerable attention since its recent endorsement by the Food and Drug Administration, as it has emerged as a promising immunotherapeutic modality within the landscape of oncology. This study explores the prognostic utility of [^18^F]Fluorodeoxyglucose positron emission tomography ([^18^F]FDG PET) in lymphoma patients undergoing CAR T-cell therapy. Through meta-analysis, pooled hazard ratio (HR) values were calculated for specific PET metrics in this context.

**Methods:**

PubMed, Scopus, and Ovid databases were explored to search for relevant topics. Dataset retrieval from inception until March 12, 2024, was carried out. The primary endpoints were impact of specific PET metrics on overall survival (OS) and progression-free survival (PFS) before and after treatment. Data from the studies were extracted for a meta-analysis using Stata 17.0.

**Results:**

Out of 27 studies identified for systematic review, 15 met the criteria for meta-analysis. Baseline OS analysis showed that total metabolic tumor volume (TMTV) had the highest HR of 2.66 (95% CI: 1.52-4.66), followed by Total-body total lesion glycolysis (TTLG) at 2.45 (95% CI: 0.98-6.08), and maximum standardized uptake values (SUVmax) at 1.30 (95% CI: 0.77-2.19). TMTV and TTLG were statistically significant (*p* < 0.0001), whereas SUVmax was not (*p* = 0.33). For PFS, TMTV again showed the highest HR at 2.65 (95% CI: 1.63-4.30), with TTLG at 2.35 (95% CI: 1.40-3.93), and SUVmax at 1.48 (95% CI: 1.08-2.04), all statistically significant (*p* ≤ 0.01). The ΔSUVmax was a significant predictor for PFS with an HR of 2.05 (95% CI: 1.13-3.69, *p* = 0.015).

**Conclusion:**

[^18^F]FDG PET parameters are valuable prognostic tools for predicting outcome of lymphoma patients undergoing CAR T-cell therapy.

## 1 Introduction

Chimeric antigen receptor (CAR) T-cell therapy represents a unique and novel form of cancer immunotherapy (1). CAR T-cell therapy diverge from standard methods by attacking tumor cells directly rather than targeting immune receptors or their interactions (2). CAR-T-cell therapy involves modifying a patient’s T-cells to express artificial receptors that target cancer-specific antigens. These modified cells will then be reintroduced to patients after adequate lymphocyte depletion. This approach combines the advantages of monoclonal antibody therapy and cytotoxic T-cells to initiate a focused immune response against cancer cells (3).

In the field of lymphoma, four CAR-T-cell therapies targeting the cluster of differentiation (CD)19 antigen on B cells have been approved by the Food and Drug Administration (FDA) and the European Medicines Agency (EMA). In 2017, two CAR-T-cell agents, axicabtagene ciloleucel and tisagenlecleucel, were approved (4, 5). The EMA granted approval for these agents the following year. Subsequently, brexucabtagene autoleucel received EMA approval in 2019 and FDA approval in 2020 (6). Finally, lisocabtagene maraleucel obtained approval from the European Medicines Agency in 2019 and the federal drug agency in 2021. Tisagenlecleucel, axicabtagene ciloleucel, and lisocabtagene maraleucel have been approved for adult patients with diffuse large B-cell lymphoma (DLBCL) who have relapsed or refractory (r/r) disease. Additionally, brexucabtagene autoleucel was approved for the treatment of adult r/r mantle cell lymphoma (7).

Positron emission tomography (PET) using [^18^F]fluorodeoxyglucose ([^18^F]FDG) is a valuable imaging technique that can be used to assess lymphoma patients who are eligible for CAR-T-cell therapy (8). This imaging tool is particularly useful for evaluating patients both before and after CAR-T-cell infusion. Typically, CAR-T-cell recipients undergo two PET scans before CAR-T-cell infusion: one at the time of decision (TD) PET/CT, which helps determine the appropriate therapeutic approach, and one at the time of transfusion (TT) PET, which is completed immediately prior to infusion. Subsequently, two PET/CT scans are performed to monitor the patient’s response to therapy: one at 1 month (M1) and the other at 3 months (M3). However, in practice, many centers adopt more flexible approaches and only obtain a single baseline PET/CT scan prior to CAR-T-cell therapy infusion (mostly at the TD timepoint) (9).

Molecular imaging using [^18^F]FDG PET has a wide range of applications beyond assessing response patterns in patients. PET-derived metrics, such as the maximum standardized uptake value (SUVmax) and its variation (ΔSUVmax) before and after CAR-T-cell therapy, along with volumetric analyses, have been used in clinical settings (10–13). Volumetric analysis commonly involves calculating the total metabolic volume (TMTV) of active lymphoma and total-body total lesion glycolysis (TTLG), which can serve as volumetric metrics at the start of treatment (i.e., baseline) and/or during M1 evaluation (14, 15).

Previous studies have demonstrated the substantial prognostic value of PET-derived metrics in various types of solid and hematological malignancies (16–21). However, the specific application of these metrics in the context of CAR-T-cell lymphoma patients remains unexplored in a meta-analysis. Consequently, our objective was to evaluate the prognostic value of metabolic [^18^F]FDG PET parameters (mainly SUVmax, TLG, and MTV) regardless of the timing, indication, or whether the values were from a single-timepoint or before-after differences. This was performed through a systematic review and meta-analysis. Pooled hazard ratio (HR) values were calculated for specific PET metrics in this context.

## 2 Methods

The methodology utilized for this systematic review and meta-analysis was based on the guidelines outlined in The Preferred Reporting Items for Systematic Review and Meta-Analysis (PRISMA) guidelines (**Supplementary File 1**). This study was not formally registered in any domain.

### 2.1 Search strategy, sourcing, and screening

Scopus, Ovid, and PubMed databases were included in our systematic literature search. The search query utilized keywords such as “positron emission tomography,” “lymphoma,” and “CAR T-cell therapy” in databases using the MeSH and Emtree systems (22). The search strategy included a combination of keywords and free text terms, without language limitations, up to March 12, 2024. Two authors, AA-I and ASA, organized and categorized the results using Microsoft Excel (version 2021). Duplicate articles were removed, abstracts were screened, and full-text articles were reviewed when abstracts lacked sufficient information.

### 2.2 Selection of eligible studies for systematic review

The research incorporated participants who satisfied distinct eligibility criteria, encompassing: a confirmed lymphoma diagnosis; receipt of CAR T-cell therapy; completion of [^18^F]FDG PET or PET/CT imaging prior to and/or subsequent to CAR T-cell therapy administration; availability of metabolic parameter data, including but not limited to SUVmax, mean SUV (SUVmean), TMTV, or TTLG; and documented survival outcomes. Exclusions applied to documents categorized as review articles, case reports, letters to the editor, editorial commentary, or conference abstracts.

### 2.3 Data collection

Two authors, AA-I and ASA, conducted a comprehensive retrieval and analysis of studies meeting the inclusion criteria for the systematic review. A new Microsoft Excel spreadsheet was created specifically for the systematic, in-depth examination of the selected articles. Pertinent data were meticulously extracted for each study, including the identity of the first author, year of publication, country of origin, research design, patient cohort size, patient sex, median age, lymphoma subtype classification, survival endpoints of the study, investigated PET parameters, and the respective timing of these measurements (baseline versus posttreatment).

### 2.4 Assessment of methodological quality and risk of bias

To assess the methodological quality of the studies selected for inclusion in the analysis, the Quality in Prognostic Studies (QUIPS) framework was utilized (23). Two authors, AA-I and ASA, independently evaluated the methodological robustness of each study, aiming to identify and mitigate any potential biases. In instances of disagreement, the authors engaged in discussions to reach a consensus. The authors visually illustrated the findings derived from the application of the QUIPS tool across its various assessment domains.

### 2.5 Criteria for meta-analysis selection

In order for studies to be considered suitable for inclusion in a subsequent meta-analysis, they must provide HR along with their corresponding 95% confidence interval (CI) values. Only those studies meeting this requirement will undergo further examination for pooled analysis. We extracted the HR with their corresponding 95% CIs from univariate Cox regression for each of the included studies in the meta-analysis.

### 2.6 Statistical analysis

Data analysis was conducted using Stata software version 17.0. This study focused on examining the prognostic impact of [^18^F]FDG PET on OS and PFS by analyzing various parameters, such as the baseline SUVmax, ΔSUVmax, TMTV, and TTLG, and their HR effect sizes. Our studies focused on implementing the results of univariate analyses since most studies included HR estimates with 95% CIs in that domain. If 95% is not listed in a study, *p*-value or number of events and sample size were implemented to render relevant 95% CI using Stata. To assess heterogeneity, the inconsistency (I^2^) index was employed, with calculations performed under the random effects model to accommodate between-study differences. An I^2^ value less than 50% indicated low to moderate heterogeneity, while values greater than 50% indicated substantial to high heterogeneity (24). Publication bias was assessed using Egger’s test. A minimum of ten studies is needed to adjust for publication bias or consider visual representation of publication bias funnel plot (25). A statistically significance threshold of *p* < 0.05 was established.

## 3 Results

### 3.1 Characteristics of studies included in systematic review

**Figure 1:**
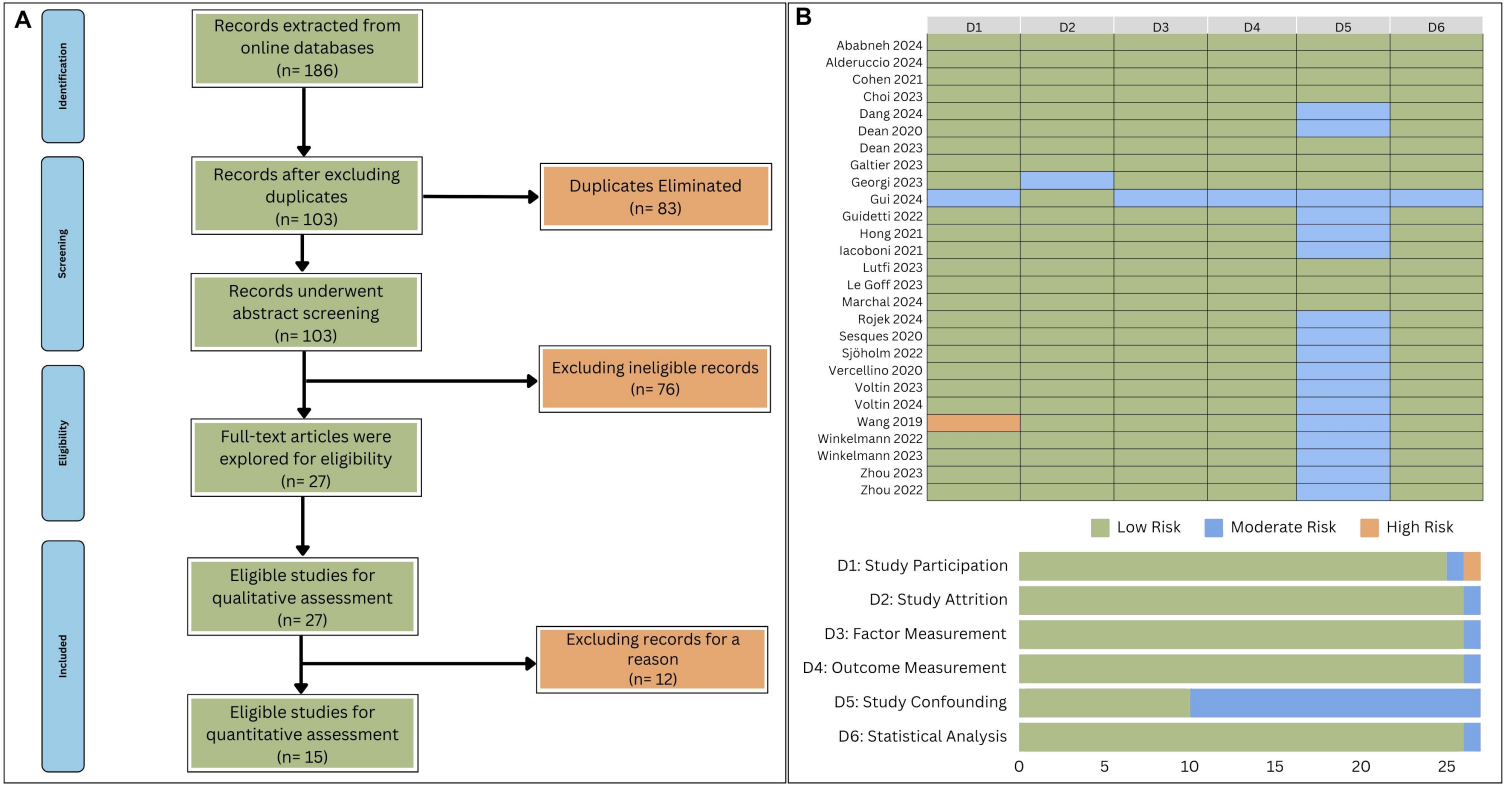
**(A)** Flowchart outlining the process of studies’ identification. **(B)** Assessment of methodological quality and bias risk using QUIPS tool.

A total of 186 academic research articles were obtained through the methodologies outlined in the preceding section. The majority of these articles were sourced from the Scopus database (*n* = 100), with fewer retrieved from Ovid (*n* = 47) and PubMed (*n* = 39). Following the removal of 83 duplicate articles, a total of 103 research papers were included in the abstract screening. The abstract screening process identified 27 eligible research articles for qualitative analysis (26–52). The PRISMA flowchart is graphically represented in **Figure 1A**. The principal characteristics and further details of eligible articles for systematic review are shown in **Table 1**.

**Table 1:** A table summarizing the key features for articles deemed suitable for inclusion in a systematic review.

Primary Author, Publication Year	Study Design	Included Patients (Male, F)	Country of Correspondence	Lymphoma Subtype
Iacoboni, 2021 (38)	Retrospective	36 (26M, 9F)	Spain	LBCL
Wang, 2019 (48)	Retrospective	19 (12M, 7F)	China	NHL
Dean, 2023 (31)	Prospective	59 (34M, 23F)	United States	LBCL
Dean, 2020 (32)	Retrospective	96 (61M, 35F)	United States	LBCL
Vercellino, 2020 (45)	Retrospective	116 (75M, 41F)	France	DLBCL
Le Goff, 2023 (39)	Retrospective	112 (68M, 44F)	France	LBCL
Guidetti, 2023 (36)	Prospective	47 (15M, 32F)	Italy	r/r LBCL
Sjöholm, 2022 (44)	Prospective	16 (9M, 7F)	Sweden	r/r LBCL
Dang, 2024 (30)	Retrospective	42 (25M, 17F)	China	DLBCL
Alderuccio, 2024 (27)	Retrospective	139 (82M, 57F)	United States	r/r DLBCL
Lutfi, 2023 (40)	Retrospective	24 (11M, 13F)	United States	LBCL
Rojek, 2024 (42)	Retrospective	61 (43M, 18F)	United States	LBCL
Sesques, 2021 (43)	Retrospective	72 (44M, 28F)	France	LBCL
Winkelmann, 2023 (49)	Prospective	62 (37M, 25F)	Germany	NHL
Cohen, 2022 (29)	Retrospective	48 (25M, 23F)	Israel	DLBCL
Gui, 2024 (35)	Retrospective	38 (23M, 15F)	China	DLBCL
Marchal, 2024 (41)	Retrospective	56 (36M, 20F)	France	LBCL
Voltin, 2024 (47)	Retrospective	88 (55M, 33F)	Germany	LBCL
Choi, 2023 (28)	Retrospective	96 (61M, 35F)	United States	DLBCL
Hong, 2021 (37)	Retrospective	41 (24M, 17F)	China	NHL
Winkelmann, 2022 (50)	Retrospective	34 (20M, 14F)	Germany	NHL
Zhou, 2022 (51)	Retrospective	24 (16M, 8F)	China	LBCL
Galtier, 2023 (33)	Retrospective	160 (99M, 61F)	France	r/r LBCL
Voltin, 2023 (46)	Retrospective	47 (29M, 18F)	Germany	LBCL
Ababneh, 2024 (26)	Retrospective	59 (33M, 26F)	United States	r/r LBCL
Georgi, 2023 (34)	Retrospective	22 (16M, 6F)	Germany	LBCL
Zhou, 2023 (52)	Retrospective	61 (37M, 24F)	China	DLBCL

M, Male; F, Female; LBCL, r/r, relapsed of refractory; Large B-cell Lymphoma; NHL, Non-Hodgkin’s Lymphoma; DLBCL, Diffuse LBCL.

The application of the QUIPS tool for assessing methodological quality and bias risk indicated that the majority of domains within all studies were characterized by a generally low risk of bias (**Figure 1B**). Seventeen studies displayed a moderate risk of bias related to study confounding, primarily due to the insufficient identification of potential confounding variables (30, 32, 35–37, 42–52). One study was noted for a moderate risk of bias in the domain of study attrition, which was ascribed to insufficient follow-up (34). Additionally, another study was marked by moderate bias in the measurement of factors, which was attributed to information bias (35). A single study was observed to have moderate bias in the measurement of outcomes, which resulted from the lack of blinded assessments (34). Regarding Study Participation, one study showed moderate bias stemming from the inadequate disclosure of participant selection criteria, and another study was identified with a high risk of bias due to defective randomization procedures (35, 48). Lastly, one study was found to have moderate bias in its statistical analysis, which was inferred to be the consequence of employing unsuitable statistical techniques (34).

### 3.2 Characteristics of studies included in meta-analysis

Of the 27 research papers examined in the systematic review, only 15 were considered appropriate for inclusion in the meta-analysis (27, 29, 32, 33, 35–39, 41, 44, 45, 47, 51, 52). These selected papers encompassed a total of 977 lymphoma patients undergoing anti-CD19 CAR T-cell therapy and underwent a thorough and detailed analysis to extract pertinent subjective data for inclusion in the meta-analysis (**Table 2**).

**Table 2:** Detailed characteristics of studies included in Meta-analysis.

#	Study Name	Sample Size	Parameters Examined	Study Design	Study Experience	Lymphoma Subtype	VOI Delineation Method	Cutoff Adaptation	Study Sample Size	Modality	Endpoint	FUD for OS (Months)	FUD for PFS (Months)
1	Alderuccio 2024 (27)	138	TMTV, SUVmax, TTLG	Prospective	Multi-Center	DLBCL	SUV 4.0	Others	Large	PET/CT	PFS, OS	14.8	6.2
2	Cohen 2021 (29)	48	SUVmax, TTLG, TMTV	Retrospective	Uni-Center	DLBCL	% SUVmax	Median	Small	PET/CT	PFS, OS	12.8	7.9
3	Dean 2020 (32)	48	TMTV	Retrospective	Uni-Center	Various	% SUVmax	Median	Small	PET/CT	PFS, OS	34.9	5.6
4	Galtier 2023 (33)	119	TMTV, Δ SUVmax	Retrospective	Multi-Center	Various	% SUVmax	ROC	Large	PET/CT	PFS, OS	22.1	4.6
5	Gui 2024 (35)	38	SUVmax, Δ SUVmax, TMTV, TTLG	Retrospective	Uni-Center	DLBCL	% SUVmax	ROC	Small	PET/CT	PFS, OS	NR	11.5
6	Guidetti 2023 (36)	47	SUVmax, TMTV, TTLG, Δ SUVmax	Prospective	Uni-Center	Various	Algorithm	Others	Small	PET/CT	PFS	NR	9.8
7	Hong 2021 (37)	41	SUVmax	Retrospective	Uni-Center	Various	Algorithm	ROC	Small	PET/CT	OS	NR	7
8	Iacoboni 2021 (38)	35	TMTV, SUVmax	Retrospective	Uni-Center	Various	% SUVmax	Others	Small	PET/CT	PFS, OS	8.2	3.4
9	Le Goff 2023 (39)	102	TMTV	Retrospective	Uni-Center	Various	Algorithm	ROC	Large	PET/CT	PFS	NR	NR
10	Marchal 2023 (41)	56	TMTV, SUVmax	Retrospective	Multi-Center	Various	% SUVmax	ROC	Large	PET/CT	PFS, OS	NR	NR
11	Sjöholm 2022 (44)	16	SUVmax, Δ SUVmax	Prospective	Uni-Center	Various	Algorithm	Median	Small	PET/MR	PFS, OS	9.3	3.9
12	Vercellino 2020 (45)	116	TMTV	Retrospective	Uni-Center	DLBCL	% SUVmax	ROC	Large	PET/CT	PFS, OS	NR	12.1
13	Voltin 2024 (47)	88	TMTV	Retrospective	Multi-Center	Various	SUV 4.0	Others	Large	PET/CT	PFS	19.2	10.3
14	Zhou 2022 (51)	24	TMTV, TTLG	Retrospective	Uni-Center	Various	% SUVmax	ROC	Small	PET/CT	PFS, OS	NR	10.7
15	Zhou 2023 (52)	61	TMTV, TTLG	Retrospective	Uni-Center	DLBCL	% SUVmax	ROC	Large	PET/CT	PFS, OS	NR	7.3

DLBCL, Diffuse Large B-cell Lymphoma; FUD, Median Follow-up Duration; NR, Not reported; PET/CT, Positron Emission Tomography/Computed Tomography; PET/MR, PET/Magnetic Resonance; ROC, Receiver operating characteristics; SUVmax, Maximum Standardized Uptake Value; TMTV, Total Metabolic Tumor Volume; TTLG, Whole-body Total Lesion Glycolysis; VOI, Volume of Interest; Δ SUVmax, Difference in SUVmax values retrieved before and after therapy.

### 3.3 Prognostic impact of baseline PET parameters on OS

**Figure 2:**
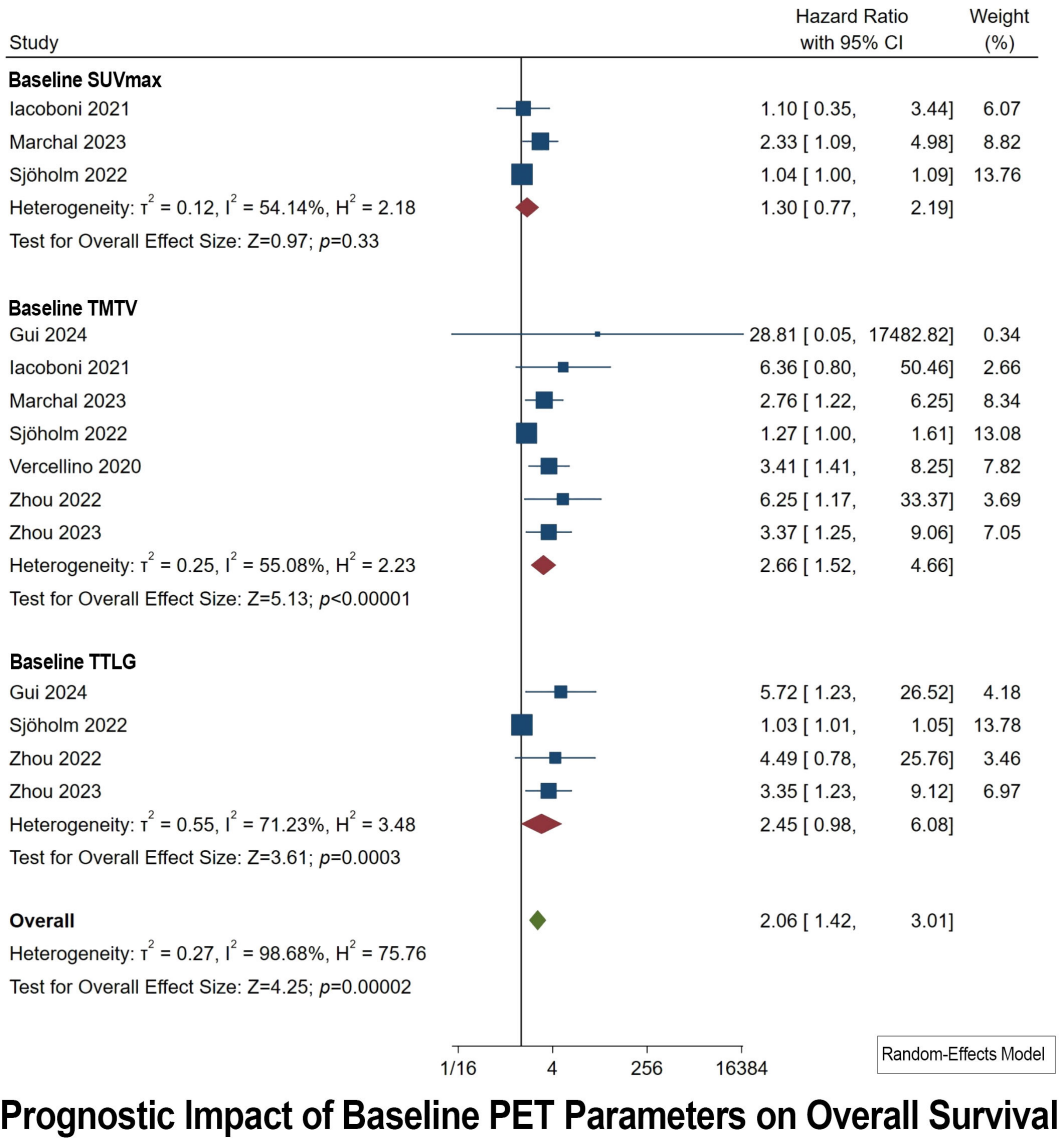
Forest plots highlighting the hazard ratios for studies exploring the prognostic value of baseline SUVmax, TMTV, and TTLG for OS.

Baseline PET Parameters were collected before initiating CAR T-cell Therapy. Overall, seven studies explored the prognostic utilities of various [^18^F]FDG PET parameters. Only three studies investigated the value of baseline SUVmax in OS prediction (35, 38, 41, 44, 45, 51, 52). Using a random model, there was no significant prognostic value of baseline SUVmax for OS (HR cutoff 1.30, 95% CI: 0.77-2.19; *p* of 0.33). Substantial I^2^ levels of 54.14% indicated a significantly high heterogeneity (**Figure 2**). Egger’s test (*p* = 0.58) revealed that there was no publication bias for recruited studies on OS.

A total of seven studies explored the prognostic value of baseline TMTV (**Figure 2**). Baseline TMTV emerged as the most powerful prognostic indicator for OS, rendering the highest pooled HR of 2.66 (95% CI: 1.52-4.66; *p* < 0.00001). Notably, there is high heterogeneity (I^2^ of 55.08%) and significant concerns about publication bias (*p* of 0.02).

A total of four studies explored the prognostic value of baseline TTLG (**Figure 2**). Baseline TTLG emerged as a statistically significant prognosticator for OS, with a second-best pooled HR of 2.45 (95% CI: 1.42–3.11; *p* of 0.00002). The result of heterogeneity was high (I^2^ of 71.23%) and publication bias was also statistically significant (*p* of 0.004).

### 3.4 Prognostic impact of post-therapeutic PET parameters on OS

**Figure 3:**
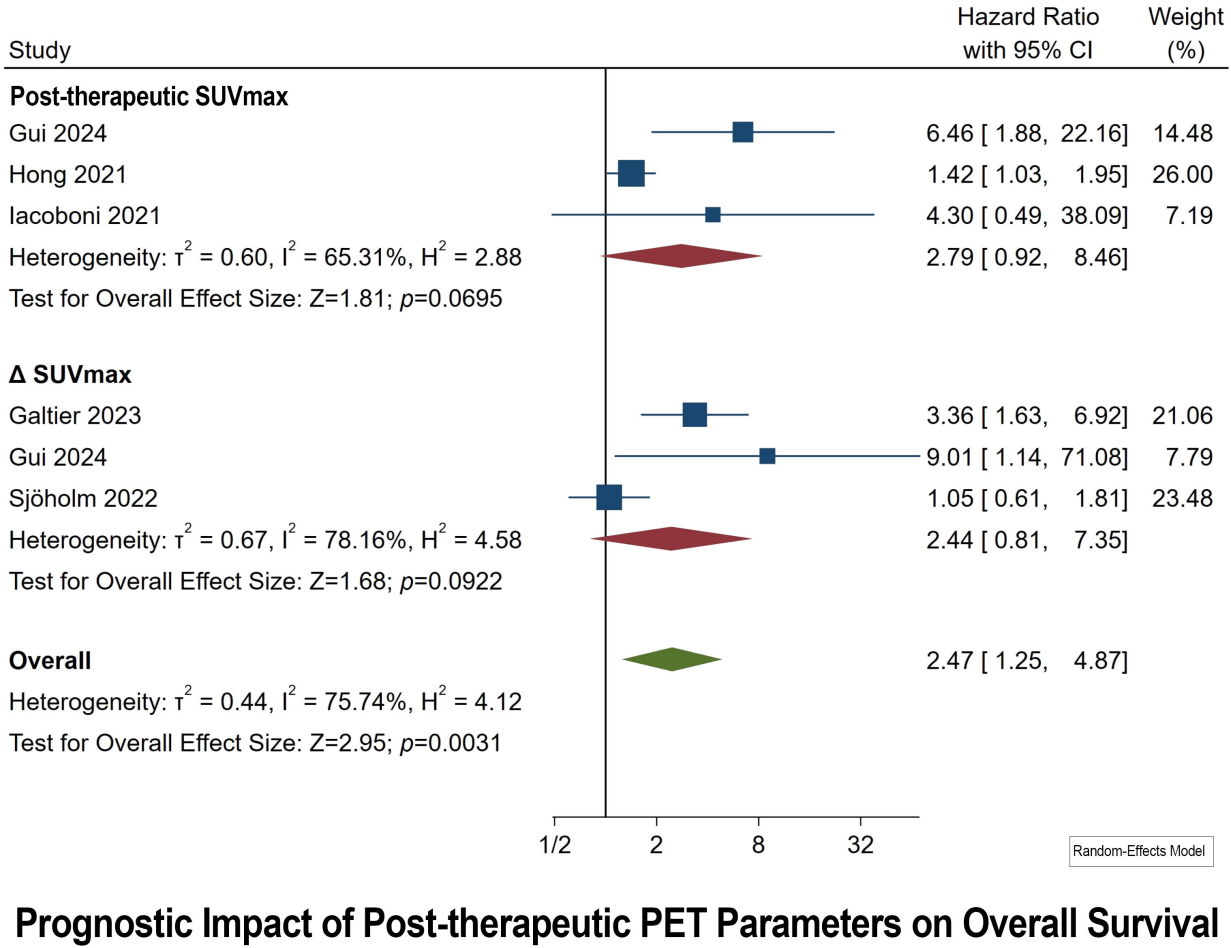
Forest plots highlighting hazard ratios for studies exploring the prognostic value of post-therapeutic SUVmax and ΔSUVmax for OS.

Post-therapeutic PET Parameters were collected after one month of CAR T-Cell infusion. In this analysis, three studies focused on the role of post-therapeutic SUVmax in predicting OS were examined (35, 37, 38). Additionally, the prognostic significance of ΔSUVmax (calculated by subtracting the values of post-therapeutic SUVmax from pretherapeutic SUVmax) was investigated in three studies (33, 35, 44). The synthesized HR analysis yielded no statistically significant outcomes, with pooled HRs for post-therapeutic SUVmax and ΔSUVmax being 2.79 (95% CI: 0.92–8.46; *p* = 0.06) and 2.44 (95% CI: 0.81–7.35; *p* = 0.09), respectively (**Figure 3**). Significant heterogeneity was observed among the studies, as indicated by I^2^ values exceeding 65%. Egger’s test excluded the presence of publication bias for both post-therapeutic SUVmax and ΔSUVmax parameters.

### 3.5 Prognostic impact of baseline PET parameters on PFS

**Figure 4:**
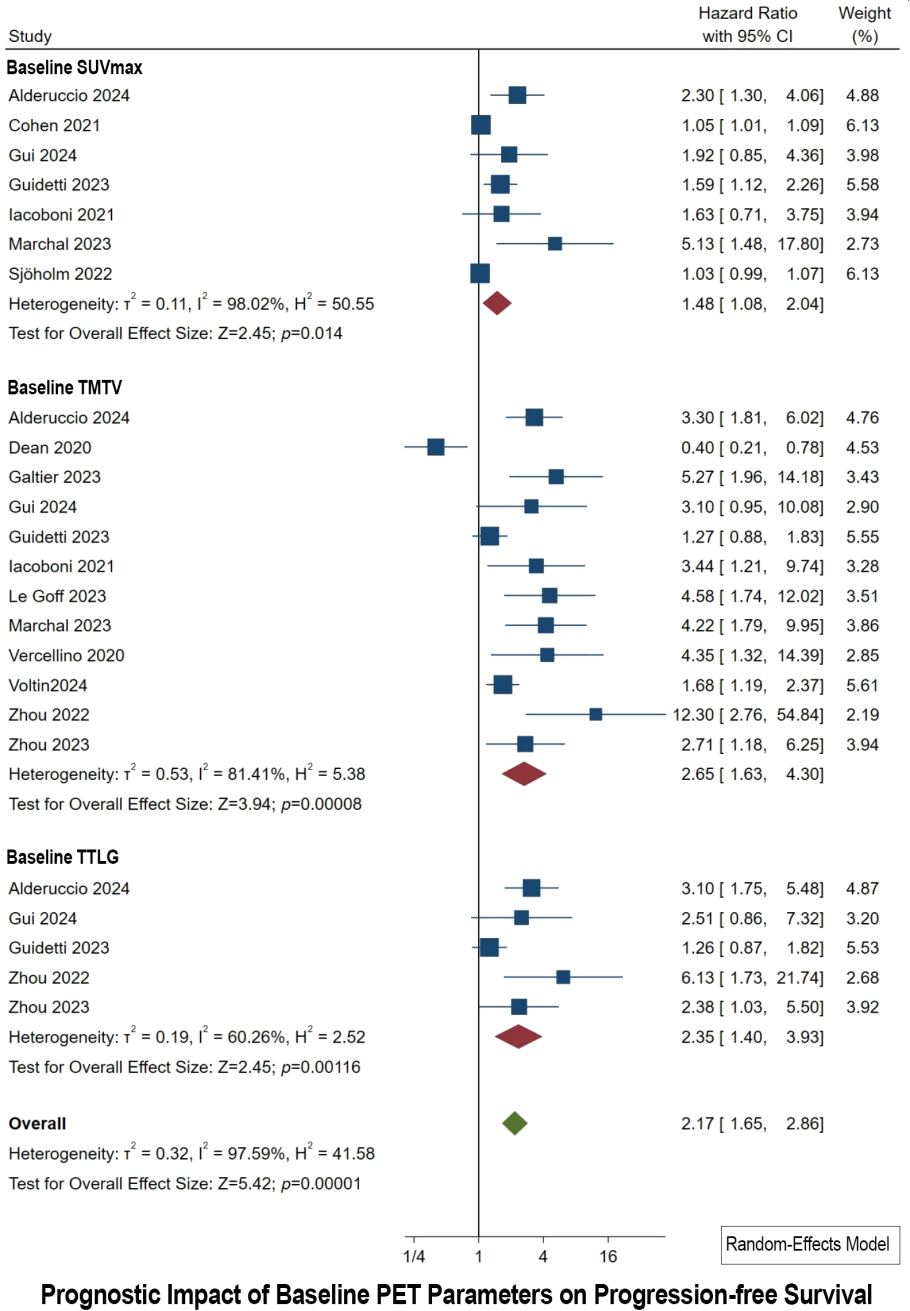
Forest plots highlighting hazard ratios for studies exploring the prognostic value of baseline SUVmax, TMTV, and TTLG for PFS.

Baseline PET Parameters were collected before initiating CAR T-cell Therapy. Concerning the SUVmax parameter, seven studies were incorporated. The HR derived from these studies varied between 1.03 and 5.13 (**Figure 4**). The aggregate HR for baseline SUVmax was calculated to be 1.48, with a 95% CI ranging from 1.08 to 2.04, thereby confirming the statistical significance of the results (*p* of 0.014). The heterogeneity observed across the studies for baseline SUVmax was quantified by an I^2^ value of 50.55%, indicating a considerable level of heterogeneity. Furthermore, Egger’s test provided evidence of publication bias (*p* < 0.0001).

For the baseline TMTV parameter, the meta-analysis included 12 studies. These studies presented HR spanning from 0.40 to 12.30 (**Figure 4**). The synthesized HR for baseline TMTV stood at 2.65, with a 95% CI of 1.63 to 4.30, underscoring the statistical significance of these findings (*p* of 0.00008). The I^2^ value for baseline TMTV reached 81.41%, suggesting pronounced heterogeneity among the studies. Additionally, Egger’s test indicated the presence of publication bias (*p* = 0.009).

Regarding the baseline TTLG parameter, the meta-analysis encompassed five studies. The individual HR for these studies ranged from 1.26 to 6.13 (**Figure 4**). The combined HR for baseline TTLG was determined to be 2.35, with a 95% CI of 1.40 to 3.93, which is statistically significant (*p* of 0.001). The heterogeneity for baseline TTLG was reported with an I^2^ value of 60.26%, denoting high heterogeneity. Contrary to the other parameters, Egger’s test for baseline TTLG did not indicate publication bias.

### 3.6 Prognostic impact of post-therapeutic PET parameters on PFS

**Figure 5:**
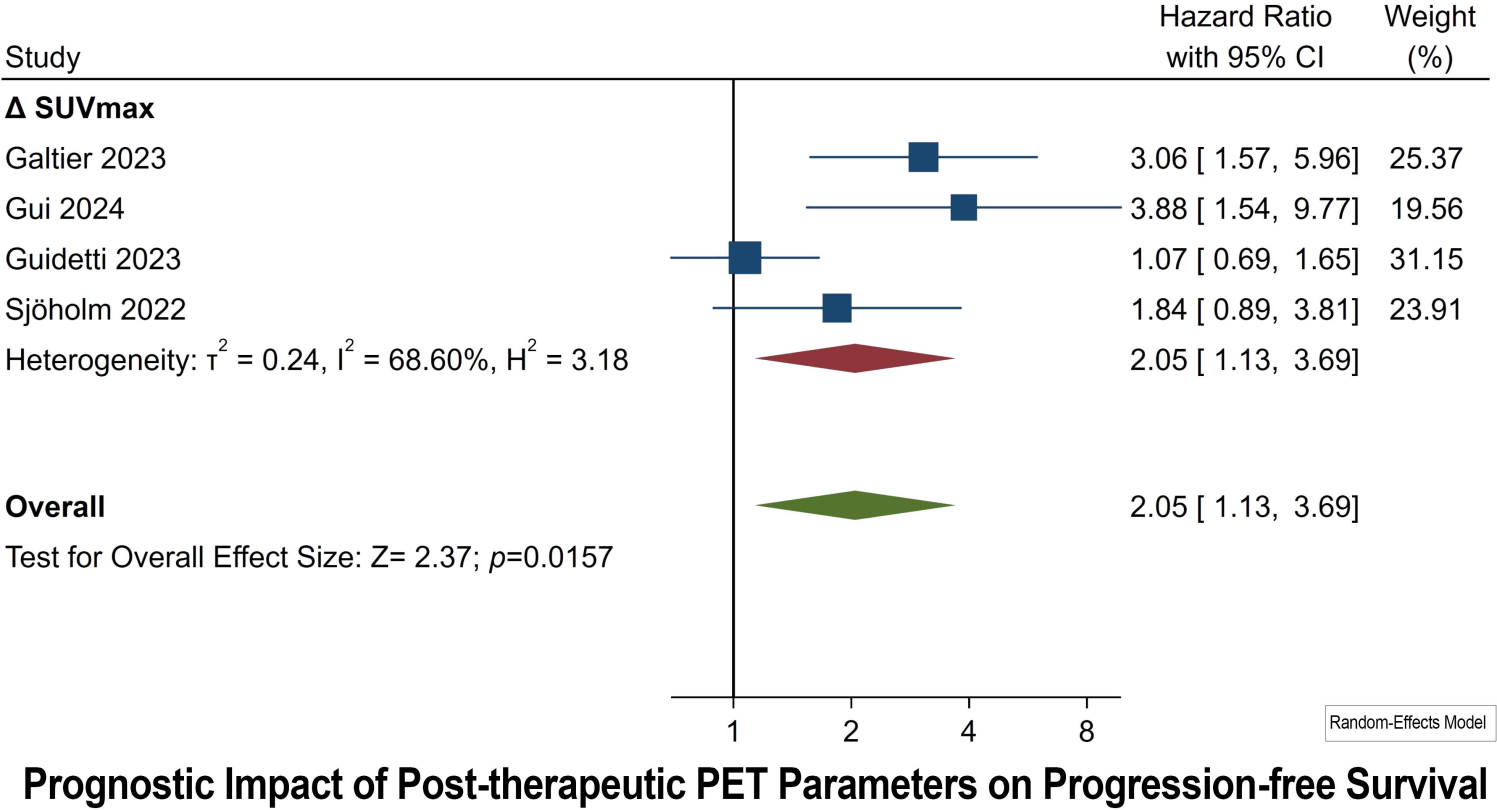
Forest plots highlighting hazard ratios for studies exploring the prognostic value of post-therapeutic ΔSUVmax for PFS.

Post-therapeutic PET Parameters were collected after one month of CAR T-Cell infusion. Four studies have been conducted to assess the prognostic significance of the ΔSUVmax parameter for PFS in post-treatment scenarios. The HRs observed in these studies vary, with the lowest being 1.07 and the highest reaching 3.88. The combined HR for the ΔSUVmax parameter, derived from all the studies, stands at 2.05, accompanied by a 95% CI of 1.13 to 3.69 (**Figure 5**). This pooled result is statistically significant, as evidenced by a *p* of 0.0157. In terms of heterogeneity, the I² statistic is calculated at 68.60%, indicating substantial variability in the effect sizes across the studies. Additionally, the Egger’s Test for publication bias yielded a significant result (*p* of 0.005).

### 3.7 TMTV: subgroup analysis

Detailed subgroup meta-analysis was conducted for TMTV parameters since it was the most encountered and examined parameter in majority of studies. This meticulously categorizes the data into different domains such as study design, study experience, lymphoma subtype, VOI delineation, cutoff adaptation, and sample size, providing a comprehensive overview of how these factors contribute to the heterogeneity and variation in HRs observed.

When it comes to OS prognostication, multicentric studies, which capture a diverse patient population and variability in imaging techniques, show a higher HR compared to unicentric studies. The analysis also revealed that patients with DLBCL have a significant association between high TMTV and OS, while the association is not significant in studies including various Non-Hodgkin Lymphomas (NHL). The cutoff adaptation method, particularly the use of Receiver Operating Characteristic (ROC) analysis, is associated with a significantly higher HR compared to the median methodology. Finally, larger sample size studies show a significant association between high HR in OS (**Table 3**).

**Table 3:** Subgroup meta-analysis for TMTV.

Overall Survival						
Domain	Factors	Number of Studies	Heterogeneity		Hazard ratio	
			(%I^2^)	*p*-value	Value (95% CI)	*p*-value
**Study Design**	Retrospective	10	81.9	< 0.00001	1.24 (1.04-1.48)	0.013
	Prospective	1	0		3.56 (1.87-6.74)	< 0.00001
**Study Experience**	Unicentric	8	81.18	< 0.00001	1.36 (0.94-1.36)	0.166
	Multicentric	3	0		3.50 (2.57-5.43)	< 0.00001
**Lymphoma Subtype**	DLBCL only	5	81.11	< 0.00001	1.32 (1.10-1.58)	0.002
	Various NHL	6	86.89	< 0.00001	1.42 (0.91-2.25)	0.117
**VOI Delineation**	% SUVmax	10	81.98	< 0.00001	1.24 (1.04-1.47)	0.013
	SUV 4.0	1	0		3.56 (1.87-6.74)	< 0.00001
**Cutoff Adaptation**	ROC	6	0		3.59 (2.34-5.52)	< 0.00001
	Median	3	88.36	< 0.00001	0.99 (0.82-1.20)	0.984
	Others	2	0		3.59 (2.34-5.52)	< 0.00001
**Sample Size**	Large (exceeding 50 participants)	7	86.03	0.054	2.05 (1.47-2.85)	< 0.00001
	Small (up to 50 participants)	4	60.89	< 0.00001	1.15 (0.94-1.39)	0.152
Progression-free Survival						
Domain	Factors	Number of Studies	Heterogeneity		Hazard ratio	
			(%I^2^)	*p*-value	Value (95% CI)	*p*-value
**Study Design**	Retrospective	11	80.8	< 0.00001	1.64 (1.20-2.24)	0.002
	Prospective	2	85.8	0.008	1.89 (1.51-2.38)	< 0.00001
**Study Experience**	Unicentric	9	82.2	< 0.00001	1.46 (1.13-1.88)	0.003
	Multicentric	4	67.5	0.026	2.29 (1.75-3.01)	< 0.00001
**Lymphoma Subtype**	DLBCL only	4	0		3.22 (2.11-4.91)	< 0.00001
	Various NHL	9	84.2	< 0.00001	1.57 (1.28-1.93)	< 0.00001
**VOI Delineation**	% SUVmax	9	83.5	< 0.00001	1.91 (1.38-2.65)	< 0.00001
	SUV 4.0	2	83.1	0.015	1.98 (1.47-2.66)	< 0.00001
	Algorithm-based	2	77.6	0.056	1.49 (1.06-2.10)	0.022
**Cutoff Adaptation**	ROC	7	0		4.19 (2.85-6.16)	< 0.00001
	Median	4	0		0.37 (0.20-0.66)	0.001
	Others	2	66.2	0.031	1.71 (1.37-2.15)	< 0.00001
**Sample Size**	Large (exceeding 50 participants)	9	83.2	< 0.00001	1.87 (1.50-2.35)	< 0.00001
	Small (up to 50 participants)	4	74.8	0.008	1.66 (1.20-2.29)	0.002

With regards to PFS, the analysis delineates insignificant statistical variation across the examined domains.

## 4 Discussion

Through this meta-analysis, we explored the prognostic value of [^18^F]FDG PET parameters and analyzed their impact on PFS and OS. Our findings revealed that both baseline TMTV and TTLG were reliable predictors of OS and PFS. Interestingly, the baseline SUVmax also emerged as a key factor for PFS stratification. Notably, the change in SUVmax (i.e., ΔSUVmax) plays a significant role in predicting PFS outcomes. This pioneering study illuminates the expanding role of [^18^F]FDG PET in evaluating the effectiveness of new cancer immunotherapies, pushing its capabilities beyond traditional response assessment.

In the context of our investigation, pretreatment TMTV and TTLG parameters demonstrated superior HR compared to the SUVmax. This prognostic superiority of TMTV and TTLG may be attributed to their comprehensive assessment of the tumor’s metabolic burden, which correlates with the tumor’s aggressiveness and the patient’s prognosis. Unlike SUVmax, which measures the most metabolically active point within the tumor, TMTV and TTLG encapsulate the metabolic diversity across the entire tumor mass, potentially leading to a more precise prognostication of treatment outcomes. Our systematic review and meta-analysis offer an exhaustive examination of the heterogeneity inherent in current studies evaluating the utility of TMTV. This includes an analysis of methodological variations, study design, investigator experience, lymphoma subtypes, VOI delineation, cutoff value adaptation, and sample size. These factors collectively contribute to the observed heterogeneity and variation in HRs, particularly in OS assessments. The findings underscore the imperative for standardized protocols and analytical methods to augment the reliability and reproducibility of cumulative research render within this domain.

To date, SUVmax is recognized as the predominant semi-quantitative index utilized in PET for the identification of lymphomatous lesions and the assessment of treatment response in a clinical setting (53). Its widespread adoption is attributed to its high reproducibility and accessibility. However, the SUVmax index is limited to providing data on a single volumetric pixel within the tumor, lacking the capacity to quantify the volume or heterogeneity of the metabolically active disease (54). Volume-based parameters such as TMTV and TTLG present advantages in assessing metabolic tumor burden. Nonetheless, debates persist regarding the optimal segmentation method for accurate measurement of TMTV and TTLG (55). Challenges in the measurement of volumetric metrics include the absence of standardization in the SUV threshold for segmentation, the suboptimal reproducibility of cutoff values, and the extensive time required for these measurements in clinical practice, as evidenced in our subgroup analysis for TMTV OS prognostication. Despite these challenges, the advent of artificial intelligence-based tools and radiomics is paving the way for overcoming these limitations (20). The development and validation of these tools have demonstrated potential in improving disease prognostication, with the ongoing development of novel radiopharmaceuticals targeting specific cellular/subcellular structures within the tumor microenvironment for diagnostic and therapeutic purposes, the field is witnessing significant advancements (51).

TMTV and lesion dissemination are emerging prognostic markers in DLBCL. TMTV, derived from [^18^F]FDG PET scans, represents the cumulative metabolic activity of all detectable lesions. Higher TMTV consistently correlates with poorer PFS and OS in DLBCL patients (56). Lesion dissemination, quantified by the maximum distance between lesions (Dmax) normalized to body surface area, also demonstrates prognostic value, with greater dissemination associated with adverse outcomes (57). While both parameters offer valuable insights, evidence suggests TMTV may have a more substantial impact on prognosis. TMTV directly reflects the tumor burden and overall metabolic activity, providing a comprehensive indicator of disease severity. In contrast, lesion dissemination offers additional context regarding disease spread but may not fully capture the tumor’s metabolic burden (8). These findings underscore the potential of TMTV and lesion dissemination as tools for risk stratification in DLBCL management (57).

Another important parameter of prognostic significance is the biochemical marker. For example, research has revealed a positive correlation between per-infusion levels of ferritin and interleukin-6 and the severity of cytokine release syndrome, a common and potentially serious complication of CAR T-cell therapy. Patients with elevated baseline interleukin-6 exhibited a higher incidence of severe cytokine release syndrome compared to those with lower levels. These findings align with observations in other hematological malignancies. For instance, in large B-cell lymphomas, elevated C-reactive protein at the time of CAR T-cell infusion has been associated with inferior outcomes (56). The prognostic value of these inflammatory markers extends beyond survival outcomes. A study examining hematologic recovery post-CAR T-cell therapy identified baseline absolute neutrophil count, hemoglobin, and interleukin-6 levels as independent factors influencing recovery (58). In the context of CAR T-cell therapy for multiple myeloma, recent research has elucidated the prognostic significance of inflammatory markers, particularly C-reactive protein, ferritin, and interleukin-6. These biomarkers, when elevated at baseline or at the at the time of CAR T-cell infusion, appear to be associated with inferior outcomes and increased toxicity. Liu et al. demonstrated that pre-infusion elevations in serum ferritin, C-reactive protein, and interleukin-6 were significantly correlated with reduced overall survival in patients with relapsed or refractory multiple myeloma undergoing CAR T-cell therapy (59).

In anticipation of future research endeavors, it is imperative to channel a more concentrated effort towards the examination of volumetric PET parameters, with a particular focus on post-therapeutic timepoints, notably M1 and subsequent intervals. Moreover, the necessity for additional prospective clinical studies becomes evident, especially those with well-defined temporal markers for the assessment of both baseline and post-therapy PET parameters. Such studies are crucial for elucidating the influence of these parameters on survival rates among lymphoma patients undergoing CAR T-cell therapy. To ensure the reliability and comparability of results across different studies, it is essential to adopt standardized protocols for [^18^F]FDG PET scanning, segmentation methods, and the determination of cut-off values. This approach will pave the way for a more cohesive and generalizable body of evidence in the foreseeable future. These studies are vital for corroborating our findings and further investigating the potential of [^18^F]FDG PET imaging in enhancing survival outcomes in a clinical setting.

In addition to these methodological considerations, the potential role of artificial intelligence in enhancing the predictive and prognostic capabilities of PET imaging in this context should not be overlooked. Artificial intelligence can be employed in prediction models for lymphoma response evaluation to CAR T-cell therapy by analyzing PET images (52). Artificial intelligent algorithms, particularly deep learning models, have shown promise in accurately classifying and predicting treatment responses based on complex imaging data (60). This suggests that artificial intelligence could play a crucial role in refining diagnostic and prognostic tools, potentially leading to more personalized and effective treatment strategies. Therefore, incorporating artificial intelligence into future research might provide valuable insights into the optimization of treatment protocols and improve survival outcomes for lymphoma patients treated with CAR T-cell therapy. While skepticism about its applicability in clinical settings remains, its potential to enhance image analysis and interpretation should be acknowledged and explored further in rigorous clinical studies.

This meta-analysis faces limitations, including a predominance of retrospective studies, significant data heterogeneity, and the inclusion of various lymphoma subtypes. It also lacks insight into PET parameters’ cut-off calculations, which would require alternative designs and approaches. Despite these challenges, it is important to recognize that this study stands as the first and only meta-analysis dedicated to this specific objective. Upon literature search, a subset of review articles were identified (8, 9, 25, 61–64), all of which were of qualitative nature (**Supplementary File 2**).

## 5 Conclusions

[^18^F]FDG PET parameters are valuable prognostic tools for assessing lymphoma patients undergoing CAR T-cell therapy. Baseline volumetric parameters, namely, the TMTV and TTLG, emerged as potential prognostic indicators for PFS and OS. Furthermore, the baseline SUVmax can predict PFS, and the ΔSUVmax can also provide prognostic insight for PFS. These findings highlight the potential of [^18^F]FDG PET as a valuable tool for predicting patient outcomes in the era of CAR T-cell immunotherapy. Therefore, [^18^F]FDG PET-metrics can function as a supplementary tool alongside other biological biomarkers for the prediction and improved management of lymphoma patients undergoing CAR T-cell therapy.

## Data Availability

The data analyzed in this study is subject to the following licenses/restrictions: Datasets are available upon reasonable request from corresponding author. Requests to access these datasets should be directed to aibraheem@khcc.jo.
